# Biotin Homeostasis and Human Disorders: Recent Findings and Perspectives

**DOI:** 10.3390/ijms25126578

**Published:** 2024-06-14

**Authors:** Chrysoula-Evangelia Karachaliou, Evangelia Livaniou

**Affiliations:** Immunopeptide Chemistry Lab, Institute of Nuclear & Radiological Sciences & Technology, Energy & Safety, National Centre for Scientific Research “Demokritos”, P.O. Box 60037, 153 10 Agia Paraskevi, Greece; xrisak15@hotmail.com

**Keywords:** biotin, biotin-(strept)avidin assays, biotin–thiamine-responsive basal ganglia disease (BTBGD), biotin-treated human disorders, biotinidase deficiency, holocarboxylase synthetase deficiency (HLCS deficiency), sodium-dependent multivitamin transporter (SMVT)

## Abstract

Biotin (vitamin B7, or vitamin H) is a water-soluble B-vitamin that functions as a cofactor for carboxylases, i.e., enzymes involved in the cellular metabolism of fatty acids and amino acids and in gluconeogenesis; moreover, as reported, biotin may be involved in gene regulation. Biotin is not synthesized by human cells, but it is found in food and is also produced by intestinal bacteria. Biotin status/homeostasis in human individuals depends on several factors, including efficiency/deficiency of the enzymes involved in biotin recycling within the human organism (biotinidase, holocarboxylase synthetase), and/or effectiveness of intestinal uptake, which is mainly accomplished through the sodium-dependent multivitamin transporter. In the last years, administration of biotin at high/“pharmacological” doses has been proposed to treat specific defects/deficiencies and human disorders, exhibiting mainly neurological and/or dermatological symptoms and including biotinidase deficiency, holocarboxylase synthetase deficiency, and biotin–thiamine-responsive basal ganglia disease. On the other hand, according to warnings of the Food and Drug Administration, USA, high biotin levels can affect clinical biotin-(strept)avidin assays and thus lead to false results during quantification of critical biomarkers. In this review article, recent findings/advancements that may offer new insight in the abovementioned research fields concerning biotin will be presented and briefly discussed.

## 1. Introduction

### 1.1. Chemical Structure of Biotin

**Figure 1:**
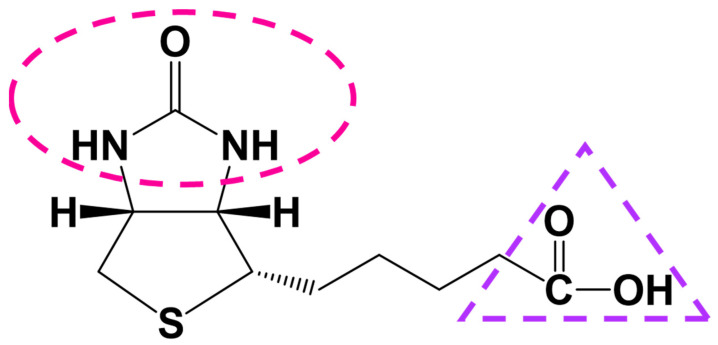
Chemical structure of d-(+)-biotin. The chemical groups involved in (i) non-covalent binding to the egg-white protein, avidin, and (ii) covalent coupling with a variety of proteins, such as the biotin-dependent apocarboxylases, are marked with an ellipse and a triangle, respectively.

Biotin, also known as vitamin B7 or vitamin H, is a member of the B-vitamin family. The biotin molecule (cis-hexahydro-2-oxo-1H-thieno [3,4-d] imidazole-4-pentanoic acid) is composed of two organic chemical rings. One of these rings, which contains a ureido group, is involved in the non-covalent binding to avidin, a glycoprotein of egg-white that exhibits an extremely high affinity for biotin. The other ring of the biotin molecule contains a tetrahydrothiophene group, to which a valeric acid moiety is attached as a side chain; through the carboxyl group of this moiety, biotin can be covalently attached to a variety of proteins and subsequently exert its cellular functions. Among all theoretically possible stereoisomers, only d-(+)-biotin is biologically active and found in nature. The chemical structure of biotin is shown in Figure 1. Biotin has a pKa of 4.5 and is quite stable in a pH range of 4–9; the vitamin is insoluble in organic solvent and poorly soluble in water (20 mg/100 mL) [1]. The extremely high affinity between biotin and avidin—as well as streptavidin, a protein of the bacterium *Streptomyces avidinii*—was the basis for developing the so-called “biotin-(strept)avidin-system”, a well-known tool that has revolutionized numerous fields of biomedicine and biotechnology, including the sensitive determination of crucial disease biomarkers through biotin-(strept)avidin-based immunoassays [2,3].

### 1.2. Biotin and Cellular Metabolism

In mammals, biotin serves as an essential cofactor of four biotin-dependent carboxylases, all of which are critical for cellular metabolism: Acetyl-CoA carboxylase [ACC, found in two distinct forms, in the cell cytosol (ACC1), or in mitochondria (ACC2)], β-methylcrotonyl-CoA carboxylase (βMCC), pyruvate carboxylase (PC), and propionyl-CoA carboxylase (PCC). ACC (E.C.6.4.1.2) is a crucial enzyme for the synthesis of fatty acids. βMCC (E.C.6.4.1.4) catalyzes the catabolism of leucine. PC (E.C.6.4.1.1) catalyzes the initial gluconeogenesis step. PCC (E.C.6.4.1.3) catabolizes the branched-chain amino acids valine, isoleucine, methionine, and threonine; the odd-chain fatty acids; and the side chain of cholesterol. Thus, biotin is an essential factor in cellular metabolism. Biotin-dependent carboxylases initially occur as apoproteins and become enzymatically active holocarboxylases after the covalent attachment of biotin via the catalytic action of the enzyme holocarboxylase synthetase (HLCS, E.C.6.3.4.10). Biotin is attached to each apocarboxylase through an amide bond formed between the carboxyl group of the valeric acid side chain of biotin and the ε-amino group of a lysine residue located in highly conserved regions of the apocarboxylases. The eminent role of biotin in cell physiology and biochemistry has been described in a series of excellent review articles [1,4,5].

### 1.3. Biotin and Gene Regulation

In last decades, it has been suggested that biotin may be involved in functions other that those associated with cellular metabolism, mainly by regulating transcription or expression of different proteins. More specifically, in addition to the well-known classical function of biotin in cellular metabolism, a distinct role associated with histone biotinylation in the cell nucleus has been attributed to the vitamin. This function may be important for cell proliferation, DNA repair, as well as regulation of the expression of a variety of genes [1]. For instance, biotin has been reported to stimulate the expression of certain proteins, such as the insulin receptor, or the human thiamine transporter-2, which might explain some of the vitamin’s roles in the treatment of human disorders (see Section 5.1.1), while it seems to suppress the expression of other proteins, such as the hepatic phosphoenolpyruvate carboxykinase [1]. As reported, biotin may affect the expression of >2000 genes in human cells [6]. Despite the currently available literature, there is still much to know concerning the exact mechanisms associating biotin with gene regulation.

Both HCLS and biotinidase, i.e., an enzyme that is also important for biotin homeostasis/recycling in humans (see Section 3.2), have been reported to play a role in the biotinylation of histones; in particular, the role of intranuclear HLCS seems to be crucial for histone biotinylation [7]. Biotinylated histones and distinct biotinylation sites have been listed in articles describing histone posttranslational modifications [8].

The number of biotinylated proteins seems to be larger than initially thought, including other proteins in addition to histones, which may associate biotin with a large series of cellular processes. Thus, as previously reported, biotinylation of heat shock proteins HSP60 and HSP72 might be linked with redox biology and immune function, respectively, while biotinylation of the two products of the *ENO1* gene, MBP-1 and ENO1, might be linked with tumor suppression and glycolysis, respectively [7].

## 2. Biotin Sources

### 2.1. Nutrition

Animal cells cannot synthesize biotin, but plant cells and microorganisms, e.g., certain bacteria and yeast, can biosynthesize the vitamin. Thus, biotin, which is necessary for human cell maintenance and homeostasis, can be obtained through diet. Biotin is widely distributed in foodstuff, e.g., organ meat (such as liver and kidney), egg yolk, some vegetables, and cow’s milk, while lean meat is considered a poorer source [1]. Biotin is also found in smaller amounts in peanuts, soybeans, sunflower seeds, mushrooms, and sweet potatoes, and at lesser amounts in some other vegetables and fruits, while the richest vegan source of biotin is nutritional yeast [9]. According to a recent article, germination, i.e., a bioprocessing approach used to increase the bioavailability of nutrients in rice, can lead to increased levels of biotin, among other dietary compounds [10]. Bioavailability of dietary biotin may vary among different foodstuffs [1].

Scientific evidence to estimate the dietary requirement for biotin is considered insufficient and, therefore, a recommended dietary allowance for biotin has not been established. However, the Food and Nutrition Board of the National Academy of Medicine, USA, has set recommendations for adequate intake (AI). The AI for adults (30 μg/day) was estimated based on the AI for infants exclusively fed with human milk. Dietary intakes of healthy adults have been reported to range from 40 to 60 μg of biotin daily, while the requirements for biotin during pregnancy may be increased [11], since biotin has been reported to be actively transported through the placenta in favor of the fetus [1,12]. On the other hand, biotin appears to be relatively nontoxic even at doses higher than 60 mg/day for several months [1].

According to the recent literature, specific population groups do not consume the recommended amount of biotin, e.g., endurance athletes [13] or groups of adolescents who consumed substantially less than the recommended amounts of fruits, vegetables, milk/dairy products, fish, and cereals/cereal products [14]. The addition of nutritional yeast or other supplementary sources of biotin might help people on a vegan diet meet their daily needs [9].

It should be noted that in the diet, biotin exists in free and protein-bound forms. Protein-bound biotin is digested by gastrointestinal proteases and peptidases to biotin–short peptide conjugates (biotinylated oligopeptides) and ε-biotinyl-lysine (biocytin); as reported, these bound forms of the vitamin are converted into free biotin prior to intestinal absorption by the action of the enzyme biotinidase [15].

### 2.2. Intestinal Microbiota

In addition to diet, another valuable source of biotin is human gut microbiota producing the vitamin, which can consequently be absorbed by the intestine through specific mechanisms [1]. Dietary biotin is processed and absorbed in the small intestine, whereas gut microbiota-generated biotin is absorbed in the large intestine [16]. The relative contribution of this source of biotin to the overall requirements of the human body for the vitamin is not well known; on the other hand, it is widely accepted that the levels of biotin produced in the large intestine highly depend on the exact composition of gut microbiota [1]. Changes in the composition of the gut microbiome and subsequent decreases in the microbial biosynthesis of biotin have been reported under certain circumstances, e.g., in people with severe obesity [17], in adolescents with type 1 diabetes [18], in patients with so-called isolated systolic hypertension [19], or in patients with primary insomnia [20]. Changes in the gut microbiota have been recently associated with a series of human disorders and comprise an extremely interesting research topic, which is considered, however, outside the main scope of the present article.

## 3. Biotin Homeostasis/Recycling

### 3.1. Biotin Uptake: The Role of the Biotin Transportation System

Valuable information concerning intestinal, renal, and liver biotin uptake can be found in earlier published review articles, e.g., in the excellent review paper by Said [1], which also describes the biotin active transport across the blood–brain barrier and the placenta. Briefly, as reported, studies have shown the involvement of a concentrative, Na^+^-dependent carrier-mediated mechanism in biotin-uptake; this mechanism is inhibited by biotin structural analogues with a free carboxyl group at the valeric acid moiety of the biotin molecule, but not by analogues with a blocked carboxyl group (as in the case of biocytin). As shown, the uptake process is under the regulation of intracellular protein kinase C and Ca^2+^/calmodulin (CaM)-mediated pathways. Once within the cell, biotin is mostly distributed among the mitochondria and cytosol fractions, while a small amount is found in the nuclei (histones) and microsomal fractions [1,21]. Biotin is excreted in the urine and feces mainly in intact form; a limited degree of catabolism to biotin sulfoxide and to bisnorbiotin may also exist [1].

Despite the so-far accumulated knowledge, investigation of the exact mechanisms of biotin absorption, trafficking, metabolism, and excretion in humans still continues to attract interest. A recent paper described the radiolabeling of biotin with a positron emission tomography (PET) radionuclide, i.e., carbon-11; the radiotracer thus developed, [^11^C]biotin, was subsequently applied to study the in vivo biotin distribution. As shown, intravenously administered [^11^C]biotin was quickly distributed to the liver, kidneys, retina, heart, and brain in rodents, while accumulation in the brown adipose tissue was observed. Orally administered [^11^C]biotin was rapidly absorbed in the small intestine and distributed to the same organs [22].

Biotin is apparently transported into the cells by a series of different transporters [23]. The main biotin transporter is believed to be the sodium-dependent multivitamin transporter (SMVT), a product of the solute carrier family-5 member-6 (*SLC5A6*) gene located on chromosome 2p23.3. SMVT is a transmembrane protein responsible for translocation of the two vitamins biotin and pantothenic acid (vitamin B5) as well as of α-lipoic acid, a vitamin-like substance; each vitamin competitively inhibits the absorption of the other two. SMVT may also be involved in the uptake of iodide (I^−^). As reported, human SMVT protein consists of 635 amino acids (predicted molecular weight: 68.6 kDa) and 12 transmembrane domains with both N- and C-termini found inside the cytosol. The protein has four N-linked glycosylation sites (at extracellular domains) and two protein kinase C-dependent phosphorylation sites (at cytoplasmic domains). SMVT is ubiquitously distributed in the human body and is most abundantly expressed in the absorptive tissues of the liver, intestine, placenta, pancreas, and kidney. Functionally, SMVT transports dissolved biomolecules such as biotin across the cell membrane against an electrochemical gradient of Na^+^. As reported, one biotin molecule is co-transported with two sodium ions in a single transport cycle [24,25,26,27].

In mammalian lymphoid tissues, biotin may also be alternatively transported by the monocarboxylate transporter, MCT1, which is located on the plasma and mitochondrial membranes and utilizes a sodium-dependent co-transport mechanism for biotin uptake into the cells. The MCT family of proteins belongs to the proton-dependent transport protein family (SLC16A), with 14 members based on sequence homology. These transporters have been reported to mediate the uptake of short-chain monocarboxylates such as lactate, α-hydroxybutyrate, pyruvate, and biotin in mammalian cells [21,27].

### 3.2. Biotin Reutilization: The Role of the Enzymes HLCS and Biotinidase

As mentioned earlier, biotin in humans acts as a coenzyme of four carboxylases, i.e., ACC, βMCC, PC, and PCC. These enzymes exist as inactive apocarboxylases, which are subsequently converted into the active holoenzymes through biotinylation that is accomplished by the action of HCLS. Upon completion of their functional tasks, the holocarboxylases undergo proteolytic degradation, which can lead to the generation of biocytin (ε-biotinyl-lysine) along with short biotinylated peptides. Finally, free biotin is released from biocytin (and/or biotinylated oligopeptides) via the action of the enzyme biotinidase and is subsequently reutilized by cells, a process that is known as the “biotin cycle” [1]. As already mentioned, biotinidase also reveals free biotin from protein-bound biotin forms that are present in food [15].

Deficient types of the HCLS enzyme may lead to a defect in the formation of biotinylated proteins, while deficient types of biotinidase may prevent the release of free biotin from protein-bound forms of the vitamin, while they may also impair biotin cellular uptake, including biotin absorption in the gut [1,15,25].

**Figure 2:**
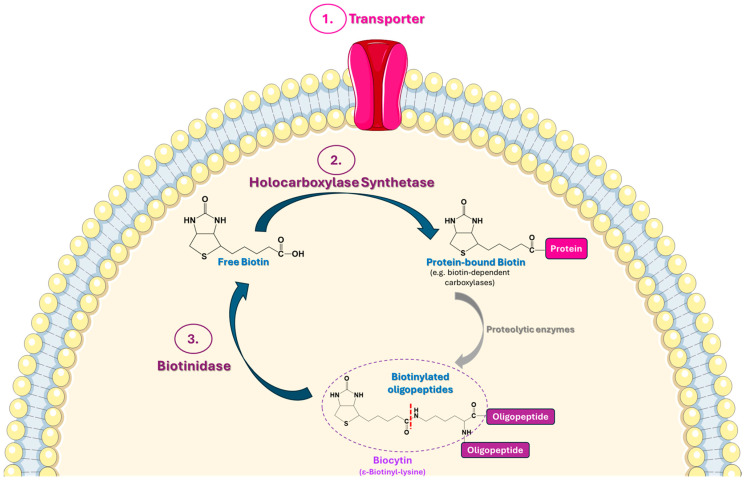
As reported, biotin enters the cells mainly through the sodium-dependent multivitamin transporter (SMVT). Inside the cell, biotin undergoes continuous recycling between its free and protein-bound forms. More specifically, intracellular biotinylated proteins are continuously formed through the action of the holocarboxylase synthetase enzyme (HLCS) and degraded to biotinylated oligopeptides and/or biocytin (ε-biotinyl-lysine), from which free biotin is finally revealed through biotinidase, which catalyzes the cleavage of the amide bond between biotin and lysine (shown with an upright, dotted red line). Overall, biotin homeostasis/recycling is mainly based on the three protein pillars shown in the figure, i.e., SMVT transporter (transporter, (1)), HLCS enzyme (holocarboxylase synthetase, (2)), and biotinidase enzyme (biotinidase, (3)).

Figure 2 summarizes the main mechanisms involved in biotin homeostasis/recycling in human cells, as described in Section 3.1 and Section 3.2.

## 4. Defects in Biotin Homeostasis/Recycling: Treatment with Biotin

Two major human genetic disorders associated with deficient activity of the enzymes HLCS and biotinidase have been thoroughly investigated so far, since they have affected a large series of individuals worldwide (especially biotinidase deficiency). Moreover, a few reports on disorder-associated variants of the gene coding for SMVT were published in last decade, which might be a topic of intensive investigation in the future.

### 4.1. Biotinidase Deficiency (BD)

BD is an autosomal recessively inherited metabolic disorder that was first described in the early 1980s [4,28]. In BD, the enzyme biotinidase is defective and biotin cannot thus be suitably recycled. Individuals with BD usually exhibit severe neurological and cutaneous abnormalities, while prompt biotin treatment can ameliorate or prevent most if not all symptoms [29].

#### 4.1.1. Symptoms

BD has been reported to affect five main organ systems: the nervous system (~67%), skin (~54%), eye (~34%), and the auditory (~27%) and respiratory systems (~18%) [30]. As reported, BD presents with neurological and cutaneous symptoms, including seizures, hypotonia, skin rash, and alopecia, usually between the second and fifth months of life. Many children have shown ataxia, developmental delay, conjunctivitis, hearing loss, and visual problems, including optic atrophy. Most but not all patients develop movement disorders. The commonest movement disorder is ataxia, followed by dystonia and cogwheel rigidity [31]. The clinical picture in early childhood is characterized by tachypnea, hypotonia, developmental delay, seizures, and cutaneous features, such as skin rash with exfoliation and alopecia, while later onsets (late infancy, juvenile age, or even adulthood) occur due to partial BD with similar but usually milder features, which may however greatly differ from case to case; thus, symptoms resembling complex forms of HSP (hereditary spastic paraplegia) have been described among cases with the delayed onset form of BD, in older children, adolescents, or even adults. Unfortunately, the age of diagnosis seems to be inversely correlated with the reversibility of BD symptoms [32,33,34].

#### 4.1.2. Screening

Diagnosis of BD can be life-saving and meets the major criteria for inclusion in newborn screening programs [29]. To this end, many countries worldwide, e.g., the USA, have included screening for BD in their national programs for newborn screening [35].

#### 4.1.3. Diagnosis by Means of Laboratory Methods (Sample Analysis)

*Enzyme activity assessment*: Diagnosis of BD is mostly based on evaluating the enzyme activity in patient blood samples [30]. More specifically, biotinidase activity is determined following the hydrolysis of either natural or artificial substrates of the enzyme by means of various systems, including initially described radioassays [36], subsequent colorimetric or fluorometric assays [35,37], or more sophisticated assays, such as tandem mass spectrometry-based ones [38]. Patients with less than 10% mean normal serum enzyme activity are classified as having profound BD and those with 10–30% mean normal serum activity are classified as having partial BD [33]. Profound BD usually results in a severe pathogenic condition. On the other hand, newborns who are affected with partial BD are mostly asymptomatic, but symptoms may appear during stressful conditions such as fasting or viral infections [35]. In some cases, the biotinidase protein levels may be assessed, in addition to or instead of the enzyme activity.

*Mutational analysis*: BD may be confirmed by means of mutational analysis of the biotinidase gene, which is located on chromosome 3p25 [30,34]. Certain enzyme mutants have been identified in affected newborns through national screening programs [39], or via independent studies [40], while more than 240 mutations have already been identified [34].

*Metabolite analysis*: BD causes late-onset biotin-responsive multiple carboxylase deficiency, which leads to acidosis or lactic acidosis, hypoglycemia, and abnormal catabolism; thus, the presence of specific toxic metabolites in serum/plasma and urine may confirm diagnosis [35,41]. For instance, deficient activity of PC results in the accumulation of lactic acid and alanine; deficient activity of PCC results in the accumulation of propionate, 3-OH propionate, and methyl citrate; while accumulation of 3-methylcrotonylglycine and 3-hydoxyisovalerate is due to the deficient βMCC enzyme activity [33].

*Combined methodology*: Many recent papers present clinical cases of BD that have been investigated/confirmed with more than one assay method [38,42,43,44].

#### 4.1.4. Incidence

The incidence of BD varies from 1:40,000 to 1:60,000 births globally. In some countries such as Saudi Arabia, the prevalence is higher due to the high consanguinity rates [33,34,35]. Thus, an incidence of 1:28,316 was reported for Saudi Arabia [45]. Moreover, an incidence of 1:25,349 was reported in an article evaluating newborn screening in the state of Mato Grosso in Brazil from 2005 to 2019 [46]. Also based on newborn screening outcome data, an incidence of 1:80,000 births and between 1:31,000 and 1:40,000 were reported for profound and partial BD, respectively, in the USA [33]. The national newborn screening program in Turkey (Republic of Türkiye) revealed high incidence; thus, based on published data from the Ministry of Health, an incidence of 1:7116 was reported [33]. According to a recent paper, blood was taken from 417,525 newborns’ heels (and stored as dried blood spots until analysis) in Diyarbakir province, Turkey, between 2011 and 2020. As a result of the diagnostic testing, 177 BD cases (incidence: 1:2359) were detected. Of the patients with BD, 56% had profound BD and 44% had partial BD. Parents of 46.6% (*n* = 55) of the patients diagnosed with BD were consanguineous and the high number of consanguineous marriages was regarded as the most important explanation for the high frequency of BD in this region of the world [47].

#### 4.1.5. Recommended Doses

Children with BD are treated with pharmacological doses of biotin (usually 5–20 mg daily) and essentially all symptoms improve clinically with biotin therapy. Seizures and movement defects usually resolve within hours to days, while the cutaneous manifestations usually resolve within weeks. Depending on the severity and frequency of the disorder episodes, many children with developmental delay can achieve improvement or regain critical development milestones they have lost [31]. Treatment for partial BD patients is still an object of debate because the biotin dosage and treatment period for this type of the disorder remain unclear; in any case, for families with a history of BD, molecular detection of putative mutants of the biotinidase gene in father and mother during pregnancy could be very helpful for the early detection of BD in a newborn and provide a better treatment strategy [35]. Newborn screening for BD should be performed in all suspected cases, as treatment with oral biotin (sometimes with doses as high as 100 mg a day) shows good results if started early in its course. However, it should be noted that even when biotin is administered precociously, response to chronic symptoms, such as deafness and optic atrophy, cannot be considered satisfactory [34]. According to a recently published systematic search including 1113 patients with BD [30], biotin treatment led to clinical stability or improvement in 89.2% of individuals, while 1.6% of reported BD individuals died due to non-availability of treatment or late diagnosis; thus, undiagnosed and non-treated BD remains a health concern [30]. Moreover, non-compliance of BD-patients with a life-long biotin treatment has been associated with serious health risks and even death [48,49].

Table 1 summarizes some of the most recently reported cases of patients with BD.

### 4.2. HLCS Deficiency

HLCS deficiency is an autosomal recessive disorder. Clinical symptoms include hypotonia, seizures, difficulties in breathing and in feeding, skin rash, and alopecia; in severe cases, developmental delay and coma may also appear. Metabolic symptoms include metabolic acidosis, organic aciduria, and hyperammonemia. Multiple mutations in the *HLCS* gene have been identified that may lead to decreased substrate affinity and impaired enzyme activity. HLCS deficiency can be diagnosed prenatally by several ways, e.g., by determining the levels of the relevant organic acids in the amniotic fluid, the activity levels of the mitochondrial carboxylases in amniocytes, or by mutational analysis. Most of the symptoms can be significantly improved with oral administration of pharmacological doses of biotin (10 mg/day) [1]. In a previously published systematic review [69], which screened 687 relevant manuscripts, 75 patients with HLCS deficiency were overall studied. As reported, most patients for whom imaging data were available exhibited abnormal features, among the most common of which were subependymal cysts, ventriculomegaly, and intraventricular hemorrhage. As proposed, HLCS deficiency should be suspected in certain cases, e.g., in children with a severe family history, or consanguineous parents, who should undergo antenatal imaging and invasive testing. Postnatal biotin supplementation might not be sufficient as a life-saving measure, since 7 of 60 patients died despite postnatal biotin administration; on the contrary, none of the patients who received antenatal and postnatal biotin died. Overall, the findings of this review [69] support the importance of identifying fetuses with HLCS deficiency as early as possible and immediately starting biotin treatment.

A recently published study described the clinical, biochemical, and molecular characteristics of Chinese patients with HLCS deficiency as well as the various mutations associated with HCLS deficiency. More specifically, 28 patients with HLCS deficiency were enrolled for the time interval 2006–2021 and their clinical and laboratory data were investigated retrospectively. Of those 28 patients, 5 patients were diagnosed through newborn screening, while 23 patients were diagnosed upon disease onset. Overall, among all patients, 24 showed symptoms such as rash, vomiting, seizures, and drowsiness at various degrees, while only 4 patients were asymptomatic. Various metabolites, including 3-hydroxyisovalerylcarnitine (C5-OH) in blood and pyruvate, and 3-hydroxypropionate, methylcitric acid, 3-hydroxyvaleric acid, and 3-methylcrotonylglycine in urine, were found at greatly increased levels among the patients. After biotin supplementation, clinical and biochemical symptoms were improved. Twelve known and six novel variants in the *HLCS* gene of patients were identified with DNA sequencing, one of which (c.1522C > T) was the most common. As proposed, newborn screening is crucial for early diagnosis, treatment, and long-lasting therapy [70].

### 4.3. Defects in the Biotin Transportation System

One case report of a child with biotin transport deficiency who responded to the administration of biotin at high doses was reported in 2002 [71]. Many years later, in 2017 [72], the case of a 15-month-old child was reported who exhibited a series of severe symptoms (failure to thrive, microcephaly and brain changes on MRI, cerebral palsy and developmental delay, variable immunodeficiency, severe gastroesophageal reflux requiring a gastrostomy tube, osteoporosis, and pathologic bone fractures). Two mutations, R94X (a premature termination) and R123L (a dysfunctional amino acid change), were identified in exon 3 of the *SLC5A6* gene of the child, i.e., the gene encoding SMVT, by means of whole-genome scanning. Functionality of the two mutants was also examined using the proper methodology and the results showed severe impairment vs. wild-type SMVT. After identification of the SLC5A6 mutations, excess biotin along with pantothenic acid and lipoate were administered to the child, with good response; as suggested, uptake of those compounds administered at high concentrations by different cells may occur via simple diffusion, thereby “overpassing”, at least partially, the defective SMVT system. This was the first report on mutations in the human *SLC5A6* gene that led to α pathologic outcome.

A few years later, in 2019, a sibling pair exhibiting early infantile-onset, progressive neurodegenerative phenotype, with symptoms of developmental delay and epileptic encephalopathy from 12 to 14 months of age was reported [73]. Using whole-exome sequencing, compound heterozygous variants were identified in SLC5A6; pathogenicity of the identified mutants was proved by suitable biotin uptake studies. One of the siblings deceased, but the second one underwent treatment with high doses of biotin, pantothenate, and lipoate, which led to great clinical improvement in the neurocognitive and neuromotor functions. This was the second report of biallelic mutations in the *SLC5A6* gene, which led to a neurodegenerative disorder due to impaired biotin, pantothenate, and lipoate uptake.

Also in 2019, the case of a 17-month-old girl who exhibited hypoglycemia and severe metabolic acidosis, leading to resuscitation, was reported [74]. According to her history, the girl had had feeding problems since birth and poor weight gain. Biochemical investigation showed increased plasma carnitine metabolites as well as persistently increased excretion of 3-OH-isovaleric acid in urine. The biochemical findings seemed compatible with BD and thus biotin supplementation was started; however, plasma biotinidase activity was only marginally decreased, which could not explain the clinical symptoms. Subsequent trio-based whole-exome sequencing revealed compound heterozygosity for variants in the *SLC5A6* gene. Thus, the dose of biotin was increased while pantothenic acid supplementation was also introduced, which led to a profound clinical improvement.

In 2022, one more case of a child with severe clinical symptoms (failure to thrive, frequent vomiting and metabolic acidosis with hypoglycemia, and mild osteopenia), who was diagnosed with SMVT deficiency due to compound heterozygous variants in *SLC5A6*, was reported. Additional genetic testing of variants of unknown significance (VUSs) along with the overall clinical improvement in the patient’s symptoms upon initiation of treatment with biotin and pantothenic acid plus lipoate supported the diagnosis [75].

Overall, all the abovementioned affected children (except the deceased sibling [73]) have received treatment since diagnosis at the following doses/administration routes: biotin (oral): 10–30 mg/day, pantothenic acid (oral): 250–500 mg/day, α-lipoic acid (oral): 150–300 mg/day [72]; biotin (i.m.) 10 mg weekly, dexpanthenol (i.m.), 250 mg weekly, α-lipoic acid (i.v.), 300 mg weekly [73]; biotin (oral), 10 mg twice a day, pantothenic acid (oral), 250 mg once a day [74]; biotin (oral): 15 mg once a day, pantothenic acid (oral): 300 mg once a day, α-lipoic acid (oral): 300 mg once a day [75].

In a recently published paper [76], the authors characterized a family diagnosed with immunodeficiency disorder presenting with low immunoglobulin levels and skin dyskeratosis. Exome sequencing revealed compound heterozygous missense variants in *SLC5A6*. As shown, the SLC5A6 variants caused biotin deficiency, which further resulted in defective B cell differentiation and antibody deficiency. Altered cellular metabolic profiles, including abnormal mitochondrial respiration and reliance on glycolysis, may explain the failure in plasma cell maturation. Replenishment of biotin improved maturation of plasma cells and helped recover the antibody-producing activity in the patient as well as in a CRISPR-Cas9 gene-edited mouse model bearing a patient-specific *SLC5A6* variant. As proposed, biotin treatment can replenish the intracellular biotin level through passive transport in patient’s B cells. Overall, the results have demonstrated that *SLC5A6* may be a causative gene for immunodeficiency, which can be treated with biotin replenishment [76].

It may be of interest to note that, to investigate defects associated with the biotin transport system, a unique, intestinal-specific, tamoxifen-inducible, conditional SMVT-knockout mouse model (SMVT-icKO) was developed a few years ago. According to subsequent studies with that mouse model, adult SMVT-icKO mice showed decreased body weight, biotin deficiency, shorter colonic length, and bloody diarrhea as compared with control mice. Also, all SMVT-icKO mice developed spontaneous intestinal inflammation associated with induction of calprotectin, proinflammatory cytokines (IL-1β, TNF-α, IFN-γ, and IL-6), and increased intestinal permeability. Furthermore, the intestines of SMVT-icKO showed activation of the NF-κB pathway, the nucleotide-binding domain, and leucine-rich repeat pyrin 3 domain (NLRP3) inflammasome. Administration of broad-spectrum antibiotics reduced lethality and led to normalization of intestinal inflammation, proinflammatory cytokines, altered mucosal integrity, and reduced expression of the NLRP3 inflammasome. Overall, the above studies show that deletion of the intestinal biotin uptake system in adult mice may lead to the development of spontaneous gut inflammation, in which the gut microbiota seems to play a critical role [16].

## 5. Other Cases of Biotin-Treated Human Disorders

### 5.1. Neurological Disorders

#### 5.1.1. Biotin–Thiamine-Responsive Basal Ganglia Disease

Biotin–thiamine-responsive basal ganglia disorder is a rare metabolic disease with autosomal recessive inheritance [77,78]. The disease was first reported as a biotin-responsive severe neurologic disease involving basal ganglia and identified in a consanguineous Saudi-Arabian family [79]. In general, the initially described patients were of Saudi, Syrian, and Yemeni origin; later reports from Europe indicated that the disorder may probably occur in all over the world [80].

As initially believed, the disease (at first called biotin-responsive basal ganglia disease) is caused by defective biotin transport across the blood–brain barrier. However, in 2005, a mutation of the *SLC19A3* gene (located in chromosome 2q36.3.) was identified, which resulted in loss or dysfunction of the thiamine transporter (thiamine transporter-2, THTR2, ThTr2, SLC19A3) [81]; interestingly, biotin is not a substrate for hTHTR2 [82]. Consequently, thiamine administration was added to the therapeutic protocols and the initial name of the disorder changed into biotin–thiamine-responsive basal ganglia disease (BTBGD) [83]. Although one study reported that a combination of biotin and thiamine was not more effective than thiamine alone [84], the regular recommended treatment of the disorder is oral supplementation of thiamine (300–900 mg/day) along with biotin (5–10 mg/kg/day), administered as early as possible and continued lifelong [77]. The initial response of patients with BTBGD to biotin seemed at first to be unexplained, but biotin responsiveness may be attributed to an increase in SLC19A3 expression through biotinylation of histones or an alternative pathway [85,86].

According to some recent review papers [31,87], BTBGD (also known as thiamine metabolism dysfunction syndrome-2, MIM: 607483) usually appears in children aged 3–10 years, although symptoms have been reported as early as at one month and as late as at 20 years of life [78]. The global prevalence of BTBGD is about 1 in 215,000 to 1,000,000 live births, i.e., BTBGD is rarer than BD [88]. Most commonly, BTBGD presents with recurrent subacute encephalopathy exhibited as confusion, seizures, ataxia, dystonia, supranuclear facial palsy, external ophthalmoplegia, and/or dysphagia, which, if left untreated, can eventually lead to coma and even death. Episodes are often triggered by fever, trauma, or surgery. Features of upper motor neuron lesion and movement disorders may also be present. Brain magnetic resonance imaging (MRI) findings are also present in affected patients. During acute crises, severe vasogenic edema can be observed; chronic changes include atrophy, necrosis, and gliosis in the affected regions. Laboratory investigations conducted mainly with gas chromatography—mass spectrometry are typically normal. The diagnosis of BTBGD is confirmed by identification of biallelic *SLC19A3* pathogenic variants [31,87].

In a recent paper, seven cases of patients with genetically diagnosed BTBGD who exhibited highly different clinical manifestations were described in detail [89]. All patients were treated with high doses of biotin (10 mg/kg/day) and thiamine (100–600 mg/day) with various clinical outcomes—except for two patients, i.e., a 4-month-old child who was treated with only thiamine (600 mg/day) and a 20-year-old individual with no clinical symptoms, who did not receive any treatment and remained asymptomatic. On the other hand, one patient, i.e., a few-day-old child, unfortunately died on day 42 of age, despite treatment with biotin and thiamine [89]. In another paper, three cases of patients with genetically diagnosed BTBGD were retrospectively presented [90]. All three patients were treated with high doses of biotin (5–100 mg/day) and thiamine (200–500 mg/day); one patient had no substantial clinical improvement, while the other two exhibited normal neurological profiles after treatment [90]. In another recent paper, nine cases of patients with genetically diagnosed BTBGD who had atypical neuroimaging findings are presented in detail [91]. All patients were treated with high doses of biotin and thiamine with various clinical outcomes, except for one patient, i.e., a 5-year-old child who was diagnosed retrospectively, did not receive any treatment with biotin/thiamine, and unfortunately died from septic shock and multiorgan dysfunction [91].

A retrospective review of the files registered in the Kuwait Medical Genetics Center for all cases with BTBGD, diagnosed clinically and radiographically and confirmed genetically, was recently published. As reported, twenty-one cases from 13 different families were diagnosed with BTBGD in Kuwait. Most cases exhibited confusion, dystonia, convulsions, or dysarthria, while three individuals were diagnosed prior to symptom onset during genetic screening. All cases received high doses of biotin and thiamine; symptoms resolved completely within 2 weeks of treatment in two-thirds of the symptomatic patients, but six of them exhibited various further symptoms, such as severe cogwheel rigidity, dystonia, and quadriparesis, probably due to delayed manifestation and management of the disease. Neuroradiological findings of the symptomatic cases revealed bilateral central changes in the basal ganglia. Two novel homozygous missense *SLC19A3* variants were found in two individuals, from Kuwait and Jordan, in addition to the previously reported Saudi founder homozygous variant [c.1264A > G; p. (Thr422Ala)], detected in the remaining cases. Age of diagnosis ranged between newborns and a 32-year-old adult (median age: 2–3 years). According to the authors, their study highlights the importance of conducting targeted molecular testing to identify the founder variant in patients with acute encephalopathy [92].

Table 2 summarizes many other recently reported cases with BTBGD.

An unusual case of encephalopathy was recently reported (“biotin–thiamine-responsive encephalopathy”). More specifically, a one-month-old boy exhibited irritability, poor feeding, and prolonged seizures; brain MRI showed multiple areas of restricted diffusion in the cerebral cortex and thalami, while the basal ganglia were not affected. After genetic testing, a homozygous pathogenic *SLC19A3* mutation was revealed and treatment with high doses of thiamine and biotin was started, which improved the infant’s alertness and development [104].

In a recent research study investigating patients with Huntington’s disease (HD) and including experimentation with HD mice, diminished ThTr2 was proposed as a pathogenic effector putatively associated with the disease, while a brain thiamine deficiency was revealed in patients with HD. As proposed, vitamin supplementation protocols similar with those used in BTBGD might be of benefit to patients with HD [105].

#### 5.1.2. The Case of Multiple Sclerosis

In the previous decade, high doses of biotin (100–300 mg/day) were tested in patients with primary or secondary progressive multiple sclerosis (MS); improved clinical outcomes were reported in nearly all participants [106]. Consequent preliminary results from multicenter double-blind placebo-controlled trials in Europe and the USA were considered promising [107]. Thus, it was reported that high-dose biotin supplementation might represent a therapeutic option in progressive MS, although the exact mechanism(s) underlying treatment remained to be clarified [108]. As proposed, the therapeutic effects of biotin on MS patients might be attributed to various biochemical functions of the vitamin, e.g., high-dose biotin might increase myelin production by increasing the generation of long-chain fatty acids (e.g., via effects on ACC1), increasing energy production via the tricarboxylic acid cycle in neuronal cells, or both [5]. It should be noted that in an earlier published paper [109], significantly lower values of biotin were found in patients with MS (both in cerebrospinal fluid and serum) in comparison with the control samples; according to that paper, the decrease observed in the biotin levels of MS patients could be attributed to intestinal malabsorption caused by the underlying disease or to putative biotin-binding immunoglobulins that might be involved in autoimmunity and MS pathogenesis. Concerning biotin dosages in MS, it is of interest to note that treatment with biotin at 10–20 mg/day (i.e., at doses similar with those used in the treatment of BD) was proposed for MS patients by Professor Barry Wolf, who also suggested that cases diagnosed as MS may actually reflect underlying BD [110,111]; however, Tourbah et al. [112] mentioned that in a pilot study [106], MS patients treated with biotin doses lower than 300 mg/day appeared to either not benefit from the treatment, or to do so to a lesser degree.

The opinion regarding the efficacy of biotin treatment in MS has dramatically changed since Cree and colleagues reported the results of a phase 3 trial (SPI2) [113]. The trial randomly assigned 642 participants with primary or secondary progressive MS receiving either biotin (through a pharmaceutical-grade formulation called “MD1003”) or placebo. The study was conducted according to the most recent clinical and MRI guidelines for investigating progressive MS. Unfortunately, as revealed, all predefined endpoints were negative. Moreover, as reported, although SPI2 indicated no safety concerns for biotin, the drug should not be considered completely harmless. Thus, a transient myopathy resembling a multiple acyl-CoA-dehydrogenase deficiency was described as an adverse event, while some studies suggested an association of new disease activity with biotin supplementation. Moreover, the possibility of false laboratory immunoassay results due to high biotin serum concentrations was reported as a risk with great possible effects in everyday clinical practice (see Section 7.1). Concluding, SPI2 showed that oral, high-dose biotin cannot be recommended as a treatment for patients with progressive MS [113,114]. Furthermore, a systematic review and meta-analysis of randomized controlled trials, including studies employing high-dose biotin (MD1003) administered orally at a dose of at least 300 mg/day and given for at least three months, was subsequently published; according to the authors, after 12 to 15 months of treatment, there was insufficient evidence that the high-dose biotin and placebo groups differed in terms of composite improvement in MS-related disability [115]. Despite the abovementioned clinical trial data, a few recent research articles still support the need to further investigate the potential of biotin treatment, alone or in combination with other compounds, in MS and other demyelinating disorders [116,117].

### 5.2. Dermatological Disorders

In a recently published paper, 14 nutritional compounds commonly used in the food or pharmaceutic industries were analyzed in terms of influencing specific skin conditions. Each of the reviewed compounds, including vitamin A, vitamin C, vitamin D, vitamin E, curcumin, chlorella, Omega-3, biotin, *Polypodium leucotomos*, *Simmondsia chinesis*, gamma oryzanol, olive leaf extract, spirulina, and astaxanthin, was reported to present some possible effects with promising benefits for specific skin conditions. According to the authors, addition of the above compounds to the diet or daily routine might have a positive influence on some skin inflammatory diseases such as atopic dermatitis or psoriasis [118].

In general, several studies have utilized biotin to treat dermatological conditions. Thus, an observational, prospective, open-label, single-cohort study on 320 children with atopic dermatitis younger than 12 years old was reported; the results suggested that supplementation with specific preparations containing multistrain synbiotics (*Lactobacillus casei*, *Bifidobacterium lactis*, *Lactobacillus rhamnosus*, *Lactobacillus plantarum*), fructooligosaccharide, galactooligosaccharide, and biotin may help to improve symptoms in children with atopic dermatitis. One stick of the preparation was administered twice a day, orally, dissolved in liquids or mixed with food, while duration of the treatment was 8 weeks; each stick (1 g) contained 7.5 mg of biotin. As reported, more than 80% of children experienced an improvement in symptoms, as measured by the Severity Scoring of Atopic Dermatitis (SCORAD) index and assessed by physicians and parents [119].

As reported in a previously published critical review of the literature, oral biotin (5 mg/day) administration in four biotin-deficient children with atopic dermatitis resulted in an improvement in xerosis and pruritus; moreover, oral treatment of various nail and hair conditions with biotin (0.9–6 mg per day) has shown some beneficial effects, according to the original papers [120]. According to another review article, biotin (2.5 to 20 mg daily) was administered to treat patients with hair loss or alopecia [121]. It should be noted, however, that, as critically mentioned, most of the studies utilizing biotin to treat dermatological disorders are small and without adequate controls [122]. Thus, several authors have reported a lack of sufficient evidence for suggesting biotin as an effective treatment for various dermatological conditions; as a consequence, several authors have proposed that biotin administration to treat/improve dermatological symptoms should be prescribed with caution, especially due to biotin interference with immunoassays determining critical biomarkers (see Section 7.1) [120,122,123,124,125]. Other authors have recommended that biotin supplementation be administered only in patients with proved biotin-associated deficiencies and that biotin levels should be measured in the patients’ follow-up [126].

## 6. Biotin Levels and/or Biotin Supplementation Proposed for Specific Population Groups: Miscellaneous Findings

### 6.1. Biotin Analysis

Reviews on analytical methods developed for determining biotin in various biological/nutritional samples have previously been reported [127,128]. Our group was among the first to develop a radioligand binding assay for determining biotin in serum samples, obtained from apparently normal subjects (100 to 840 ng/L, mean 340 ng/L), pregnant women (100–300 ng/L), as well as patients undergoing chronic hemodialysis (0.5–3.0 μg/L) [129]. An ELISA-type assay was also developed by our group [130]. ELISA kits for biotin are currently commercially available and have been widely used for many years [76,126,131,132]. In a recent paper, biotin (among other analytes) was measured in blood plasma of healthy volunteers living in a low-altitude area who had been transported to high altitude using ultrahigh-performance liquid chromatography—tandem mass spectrometry (UHPLC-MS/MS) [133]. New analytical methods have also been described in the recent literature, including an artificial intelligence-aided, competitive massive parallel spectroscopy (MPS) bioaffinity assay [134], a chemiluminescent binding assay based on avidin and biotinylated quinone [135], an impedimetric immunosensor assay [136], a competitive immunoassay based on magnetic beads and gold nanoparticle probes [137], a method based on column-switching liquid chromatography–tandem mass spectrometry [138], as well as two types of CRISPR/Cas12a-powered competitive immunoassays [139,140].

### 6.2. Biotin Levels in Specific Population Groups

Suboptimal biotin levels have been reported for specific population groups [1], although it is not always clear whether measurement of biotin in specific samples was conducted or suboptimal biotin was indirectly assumed; several cases/conditions associated with a low biotin status have attracted special attention and some continue to be mentioned in the recent literature, e.g., sudden infant death syndrome [141], long-term parenteral nutrition [142,143], MS and/or treatment with anti-epileptic drugs [1,109], elevated inflammation [23,144], or pregnancy [145,146]. Interestingly, according to a recent review article, the strength of recommendation to provide supplementary biotin during pregnancy is characterized as “weak” [147]. Moreover, a recently published study investigated whether there is a difference in biotin levels between pregnant women with and without gestational diabetes mellitus (GDM). As revealed, biotin levels were slightly decreased in pregnant women with GDM as compared with control pregnant women, though the difference was not statistically significant [132].

In another recent study [126], one hundred and five patients of a dermatology outpatient clinic had their biotin levels determined, while the patients’ files were retrospectively analyzed. As revealed, biotin is not significantly correlated with hemogram parameters, while there was a moderately negative relationship between biotin levels and triglyceride levels. The mean biotin level in all dermatology patients was 311.18 ± 158.79 ng/L. Biotin levels detected in 27 patients (25.7%) were assessed as deficient, in 48 patients (45.7%) as suboptimal, and in 30 patients (28.6%) as optimal. No statistically significant differences in biotin levels between genders were found (which agrees with previous reports [148]). Moreover, it was observed that biotin levels increased with age, but the differences found could not be considered statistically significant. In addition, although there was only one case, biotin levels were found to be low in a patient with erlotinib-induced skin rash [126].

In another study, the authors investigated the relationship between biotin metabolism and human allergic sensitization or allergic diseases; more specifically, serum levels of biotin, total immunoglobulin E (IgE), and allergen specific IgEs were determined in more than 400 Japanese schoolchildren aged from 6 to 12 years. As revealed, mean serum biotin levels in children with cedar pollinosis, but not with other allergic diseases such as asthma and allergic rhinitis, were significantly higher than in those without, which suggests that a correlation exists between serum biotin levels and the development of cedar pollinosis. Further prospective studies are needed to evaluate any causal relationship between biotin metabolism and cedar pollen sensitization and pollinosis development [149].

As earlier reported, patients with type 2 diabetes have demonstrated lower circulating levels of biotin compared to healthy controls [150]. In a recent paper in the field of obesity [17], the authors performed metagenomic analyses in 1545 subjects from the MetaCardis cohorts along with various experiments in mice (e.g., fecal microbiota transfer, bariatric surgery, and supplementation with biotin and prebiotics)—including germ-free and antibiotic-treated animals. As revealed, severe obesity is associated with an absolute deficiency in biotin producers and transporters among gut bacteria, abundances of which seem to depend on host metabolic and inflammatory conditions. Suboptimal circulating biotin levels have been found in severe obesity along with altered expression of biotin-associated genes in human adipose tissue. Supplementation of mice fed with a high-fat diet with fructo-oligosaccharides and biotin improved their microbiome diversity as well as the potential for bacterial production of biotin, while also limiting weight gain and glycemic deterioration. As the authors concluded, supplements combining biotin and prebiotics might help prevent metabolic problems in severe obesity.

### 6.3. Biotin Supplementation Proposed for Specific Population Groups

As suggested in a very recently published paper [151], dental management of patients who exhibit oral manifestations of mucus membrane pemphigoid (MMP), an autoimmune-based bullous disease affecting the mucous membranes, may involve the use of vitamin supplements and probiotics. More specifically, administration of an oral probiotic (one tablet/day for 90 days) that contained *Bifidobacterium lactis* HN019, *Kluyveromyces marxianus fragilis* B0399, colostrum, and biotin was reported in this study. According to the results reported, two patients out of thirteen studied (15.4%) had no benefit from therapy, four patients (30.8%) had a partial response to treatment, and seven patients (53.8%) had a complete response to treatment.

In another recent study, the authors reported plasma p-tau181 and glial fibrillary acidic protein (GFAP) levels in community-dwelling older adults who had received supplementation with a nutritional blend including biotin (0.15 mg/day); an increase was observed in both levels, but no difference was found between subjects receiving the nutritional supplement for one year and those receiving placebo. Nevertheless, according to the authors, further investigation is necessary to clarify whether nutritional supplementation may be able to protect brain health and cognitive functions and prevent Alzheimer’s Disease by affecting tau accumulation, amyloidosis, and glial activation [152].

Based on previously published theoretical considerations and information from the literature on biochemical pathways that may be involved in the effects of biotin supplementation [153,154], a recent paper proposed that high-dose biotin may mimic and possibly potentiate the activating impact of nitric oxide on soluble guanylate cyclase and can act as an activator of the latter. To this end, the authors speculated that high-doses of biotin (10 mg, 2–3 times a day) may have practical potential for use in asthma management; however, according to the authors, biotin should first be tested in rodent models of asthma or other lung disorders [155]. In a related study, the authors proposed that supplementation of biotin at high doses (10–20 mg, twice daily) can directly activate soluble guanylate cyclase (although not more than 2–3-fold) and thus boost guanosine 3′,5′-cyclic monophosphate (cGMP) levels in mast cells, mimicking the physiological effects of nitric oxide and carbon monoxide in that regard; according to the authors, the above effect might have clinical potential for mast cell stabilization and allergy control, although lack of in vivo preclinical data is a limitation of the study [156].

A randomized double-blind controlled study was conducted to evaluate the effect of probiotic treatment in individuals with a major depressive disorder diagnosis. More specifically, within an inpatient care setup, 82 currently depressed individuals were randomly assigned to receive a treatment of either a multistrain probiotic plus biotin or biotin plus placebo for 28 days. Clinical symptoms along with gut microbiome were analyzed at the beginning of the study, after one week and after four weeks of treatment. As revealed, four-week probiotic plus biotin supplementation led to an overall beneficial effect, especially in the diversity profile of the gut microbiota, which is suspected to affect brain functions and behavior as well as decrease inflammation status [157].

A study on the nutritional status of patients with phenylketonuria in Japan that was published in the previous decade reported low biotin levels in urine and recommended the addition of biotin (and selenium) to phenylalanine-free milk [158]; it is interesting to note that an even earlier study had reported impairment of biotin recycling in children with phenylketonuria who exhibited seborrheic dermatitis [159].

In a study employing animal experimentation, colitis was experimentally induced in mice through treatment with dextran sodium sulfate (preclinical model for inflammatory bowel disease, IBD); as shown, biotin oral administration to these animals proved beneficial, since it led to delayed onset and reduced severity of colitis while also accelerating healing. As the authors reported, biotin exerts its beneficial action by reducing the activation of NF-κB, which prevents the production of inflammatory cytokines and helps maintain the integrity of the intestinal barrier. Since the NF-κB pathway is important in the development of IBD, the findings of the study suggest that biotin may have therapeutic potential for IBD patients [160].

As previously reported in another animal-based study, an increase in the colonic concentration of biotin, which was achieved through feeding mice with a biotin-rich diet, led to significantly reduced enterohemorrhagic *Escherichia coli* adherence (EHEC); according to the authors, this finding might indicate that biotin supplementation could be useful to prevent EHEC infection in humans as well [161]. A recent related study reported that supplementation with probiotics containing biotin-excreting microbiota strains may offer a more successful approach to preventing EHEC, since oral consumption of biotin may not sufficiently increase colonic biotin levels, due to absorption of the vitamin in the small intestine [162].

## 7. Further Perspectives

### 7.1. Treatment with High Doses of Biotin: Its Effects on the Results of Biotin-(Strept)avidin-Based Immunoassays

In 2017, the Food and Drug Administration (FDA), USA, issued a warning according to which biotin may interfere with laboratory immunoassay tests determining various disease biomarkers, including troponins and thyroid-stimulating and parathyroid hormones. More specifically, since almost all these immunoassays are based on the biotin-(strept)avidin technology, high biotin levels in the patient’s sample may cause falsely elevated or reduced measurements for the biomarker of interest. In 2019, the FDA updated the safety warning of 2017 [122].

Immunoassays that are based on biotin-(strept)avidin technology are widely used for measuring various biomarkers in patients’ biological samples and are very common in high-throughput analytical platforms employing automatic analyzers [2]. Erroneous analytical results of biotin-(strept)avidin-based immunoassays have mostly been associated with patients’ supplementation with high doses of biotin [5]. Biotin interference may result in either falsely increased or falsely decreased concentration values of the biomarker of interest, depending on the assay design. Thus, elevated concentrations of biotin in the biological sample may result in falsely increased analyte/biomarker concentration if a competitive immunoassay is used for the determination of the latter (positive interference); on the contrary, biotin shows negative interference if a sandwich immunoassay format is used [5,124]. It should be noted that, as reported, the artifacts of two distinct hormone assays in the same sample can resemble an apparently coherent clinical profile; e.g., falsely increased free thyroxin (T4) and falsely decreased thyroid-stimulating hormone (TSH) in the same blood sample may be interpreted as hyperthyroidism [5].

It is of utmost importance that patients and/or their families always let any healthcare professionals clearly know that they take pharmacological doses of biotin. On the other hand, the laboratories must know if a sample has been obtained from an individual under treatment with a high dose of biotin. As reported, the FDA has recommended discontinuing biotin treatment for 8 h before sampling for patients taking biotin at doses of 10 mg/day, 3 days for 100–300 mg/day, 7 days for children taking 2 and 15 mg/kg/daily, and inform the laboratory if a diagnostic test was performed while the patient was still under biotin treatment, since it is important to discuss the interpretation of the results in the light of the possible interference of biotin [122].

Despite the abovementioned warnings and the relevant publications, several recent papers have described cases of false immunoassay results caused by high-dose biotin intake. Thus, in a patient with Graves’ disease, probably due to high biotin intake, elevated free T4 and free triiodothyronine (T3) were measured, which were at first falsely attributed to an aggravation of the disease [163]. Elevated free T3 and free T4 and low TSH were measured in a patient with MS due to treatment with high doses of biotin; since measured values of hormone levels resembled hyperthyroidism, anti-thyroid medication was initially administered to the patient [164]. Biotin supplementation can also lead to falsely decreased urinary human chorionic gonadotropin (hCG) values; false results may be of high significance, e.g., in antidoping testing [165].

Anti-biotin immunoglobulins that are present in the serum of some human individuals (in approximately 3% of normal adults) may also cause false results in biotin-(strept)avidin-based immunoassays, as previously reported [166].

### 7.2. Biotin and Cancer

According to the previous literature, the ability of biotin to de-repress epigenetically silenced genes in cancer cells and activate these genes in normal cells might have important implications for cancer prevention and therapy [167]. On the other hand, several reports in the literature have indicated that cancer cells could import more vitamin compared to normal cells, possibly for maintaining high proliferative status [168], which has been correlated with tumor aggressiveness and poor outcomes [169].

The main transporter for biotin, SMVT, is overexpressed in various aggressive cancer cell lines, while overexpression of SMVT has been found higher than that of the folate receptor; therefore, biotin demand seems to be higher in rapidly growing tumors than in normal tissues [170].

Human SMVT was proposed to serve as a novel diagnostic and prognostic marker for gastric cancer, exhibiting increased levels in cancer cells compared with their non-cancerous neighboring cells, which moreover correlated negatively with patients’ survival [171]. Furthermore, as later reported, SMVT expression is induced in Ras tumorous midguts, while silencing of the gene reduces tumor size and mitosis. In addition, changes in biotin levels, through dietary or microbiome manipulation, may also affect tumorigenesis [172].

Several biotin-conjugated compounds have been reported in the literature and applied to the specific targeting of tumor cells for cancer diagnosis and/or for selective delivery of therapeutic agents in tumors [170]. For instance, biotin, as a tumor-targeting agent, and curcumin, as a potential carrier of ^68^Ga, were immobilized on nano-chitosan, and a novel bio-nanocomposite was designed (Chit/Cur@Biot). As shown with various in vitro studies, Chit/Cur and Chit/Cur@Biot bio-nanocomposites exhibit antitumor activity, while the Chit/Cur@Biot bio-nanocomposite is more effective than Chit/Cur against cancer cell lines at high concentrations; moreover, Chit/Cur@Biot exhibited higher cellular uptake by cancer cells [173]. Furthermore, a biotinylated cyanine derivate with a phenol group available for ^131^I-labeling was developed, which showed great potential for near-infrared imaging and targeted radionuclide therapy of tumors [174]. Another recent study described the SMVT-mediated delivery of lysozyme/protein cargo by a formulation composed of non-covalent protein complexes with a mixture of biotin-conjugated polyethylene glycol (PEG)–poly-glutamic acid (GA) as targeting and complexing functionalities; the so-designed complexes achieved intracellular delivery of the cargo/biotherapeutic in lung-derived A549 epithelial cells in vitro [175]. In another recently published paper, the authors designed multi-functional nanoparticles (MFNPs) that carried biotin (among other specific compounds, each serving a specific purpose) to enter the cancer cells; an in vitro inhibition experiment and in vivo antitumor therapy showed that the MFNPs could be employed as an excellent carrier to treat hepatocellular carcinoma [176]. In another recent study, a novel near-infrared tracer conjugated to coenzyme-R (i.e., vitamin B7/biotin) was preclinically evaluated for the detection of metabolically active cancerous cells; as revealed, the so-called (CR)-S0456 novel tracer specifically labels highly proliferative tumors through SMVT transport channels and might be further evaluated in human clinical trials for intraoperative molecular imaging-guided resections [169]. It should be mentioned here that in a recently published article [27], the authors interestingly drew attention to the previous finding that the free carboxyl group of biotin plays a crucial role in the SMVT-mediated uptake of the molecule, while chemical modification, e.g., to an amide or ester group (as it occurs in most of the biotin conjugates targeting SMVT), has been reported to prevent uptake. This apparent controversy indicates, according to the authors, that further studies are necessary to elucidate the exact cellular uptake mechanisms of biotin conjugates [27].

In addition to the biotin transporter, SMVT, the two crucial enzymes involved in biotin homeostasis/recycling, i.e., biotinidase and HLCS, have been associated with cancer. Thus, biotinidase was previously proposed to be a breast cancer biomarker through differential profiling of the breast cancer plasma proteome [177]. Moreover, HLCS expression was immunohistochemically investigated in breast tissue samples obtained from 65 Thai patients, while the role of HLCS in supporting invasion was investigated in *HLCS* knockdown MCF-7 cells; as revealed, overexpression of HLCS was significantly associated with metastasis of breast cancer cells to other lymph nodes but not the sentinel and axillary lymph nodes, as well as with reduced survival time of patients with breast cancer [178]. In a paper concerning glioblastoma, sulconazole (SN), i.e., a compound with anti-glioma stem cell (GSC) properties, was reported to disrupt biotin distribution among carboxylases and histones, while transcriptomic and metabolomic studies of SN-treated GSCs revealed changes characterizing biotin-deficient cells; moreover, SN treatment reduced histone biotinylation/acetylation, while *HLCS* silencing impaired GSC tumorigenicity in an orthotopic xenograft brain tumor model, indicating poor prognosis of high HLCS expression in glioblastoma [179]. In a very recent study, the biochemical changes associated with *HLCS* knockdown in MDA-MB-231 cells were investigated to further elucidate the pathways involving HLCS in breast cancer cells [180].

### 7.3. Miscellaneous Recent Research Results Related with Biotin

According to a recent study, upregulated biotinidase levels were found in idiopathic membranous nephropathy (IMN), i.e., a pathologically defined disorder of the glomerulus, which is primarily responsible for nephrotic syndromes (NSs) in nondiabetic adults. According to the authors, theirs was the first study correlating biotinidase and IMN, and the finding might deserve further investigation [181]. In another recent study, by employing drug database screening and AutoDock validation, the authors proposed that prostaglandin, valsartan, “biotin A”, luteolin, and curcumin might be potential drugs for antagonizing the plasminogen activator, urokinase receptor (PLAUR), which was identified as an effective diagnostic and possible therapeutic marker for atherosclerosis lesion progression [182]. In a very recent article, the authors employed ovariectomized (OVX) rats as an in vivo model to characterize the anti-osteoporotic activity and metabolic mechanism of the ethanol extract of *C. deserticola* (CHE), a Chinese traditional medicinal plant. CHE treatment demonstrated significant anti-osteoporotic activity. The main interventions of CHE on OVX rats involved the modulation of several key pathways, including biotin metabolism, as shown by biotin downregulation [183]. As previously reported, biotin can enhance the sperm motility and prolong the survival of frozen–thawed semen samples, which might be beneficial in the assisted reproductive technology field [184]. In a recent article, the authors showed that intraperitoneal administration of biotin to mice resulted in an increase in testosterone levels in serum and testes; as reported, this effect might be mediated by an increase in adenosine 3′,5′-cyclic monophosphate (cAMP) and subsequent activation of protein kinase A [185]. In another recent article, it was implied that biotin, among other pharmacologically active compounds (ascorbic acid, biotin, caffeine, and L-cysteine) may target COVID-19-related differentially expressed genes and affect COVID-19-associated male infertility [186]. According to a recent study, secretion of positive emotional tears was correlated with biotin and caffeine metabolism, which might be exploited in the identification of fake tears [187].

## 8. Concluding Remarks

Biotin (Figure 1) is of utmost importance for cell biochemistry and physiology. Its role in cellular metabolism as a cofactor of the biotin-dependent carboxylases has been well established for many years, while its apparent involvement in gene regulation is also of high interest and continues to be investigated. Biotin is not synthesized by human cells, but it is found in the diet and is also synthesized by the human gut bacteria, although contribution of the latter source to the overall vitamin intake cannot be precisely estimated. The levels of biotin produced in the large intestine are highly dependent on the exact composition of gut microbiota, which might change under certain circumstances/disorders. According to the previous and recent literature, changes in the intestinal microbiome that may influence/be influenced by bacterially produced biotin in health and disease states is a very interesting topic that deserves thorough future investigation.

Biotin homeostasis/recycling depends on several factors, among which the following are considered of principal importance: (i) effectiveness of intestinal uptake of biotin, which is mainly based on the sodium-dependent multivitamin transporter, SMVT, and (ii) effectiveness of biotin reutilization within the human organism, which is mainly based on the enzymes HLCS and biotinidase (Figure 2).

The adequate intake for biotin is 30 μg/day for adults, which is easily achieved through a well-balanced diet under normal conditions. However, under certain circumstances, especially in the case of BD and HLCS deficiency, administration of high (”pharmacological”) doses of biotin has been recommended (usually 5–20 mg per day, but sometimes higher, e.g., 200 mg per day) and biotin treatment has often proven life-saving. The wide range of clinical symptoms, patient ages, and responses to biotin treatment (BD, Table 1) indicate that there is still much to learn even about well-established biotin-related deficiencies, such as BD and HLCS deficiency.

Some very interesting recent papers have described a few distinct clinical cases of children mainly exhibiting a series of severe neurological symptoms that often resemble those of BD and are apparently due to defective mutants of the biotin transporter, SMVT; high doses of biotin (orally: 10–30 mg per day; i.m.: 10 mg weekly) along with pantothenic acid and, in most cases, α-lipoic acid have been administered to children as a therapeutic treatment. Thorough investigation concerning the genetic background of similar clinical cases that might appear in the future will shed more light on this group of human disorders.

Among neurological disorders, the so-called biotin–thiamine-responsive ganglia disease (BTBGD) has been treated with high doses of biotin. As is now well known, BTBGD is associated with defects in the thiamine transporter; since the expression of the latter might be affected by biotin, both biotin (5–100 mg per day) and thiamine are recommended for the treatment of BTBGD patients in almost all therapeutic regimens, often with life-saving effects. The wide range of clinical symptoms, patient ages, and responses to therapy (Table 2), along with the yet not fully elucidated role of biotin in treatment protocols, indicate that there is still much place for enriching our knowledge about this biotin–thiamine-treatable disorder.

A special case of neurological disorder that was proposed to be treated with high doses of biotin (apparently the highest ones used so far, e.g., 100–300 mg per day) is MS, although the exact mechanism(s) underlying treatment was not fully clarified. However, despite the encouraging preliminary findings, the results of recent meticulous clinical trials concluded that high-dose orally administered biotin cannot be recommended for patients with progressive MS; nevertheless, some recent articles still support that biotin supplementation in MS and/or other demyelinating disorders needs to be further investigated.

It should be noted that the FDA has warned clinicians and patients that high biotin levels can affect clinical biotin-(strept)avidin assays and thus lead to false results during quantification of critical disease biomarkers; problems associated with such false assessment continue to appear, according to the recent literature. On the other hand, specific research areas, e.g., concerning overexpression of SMVT and/or the enzymes biotinidase and HLCS in various cancer cells, should be further investigated and clarified. Keeping the above in mind, one could suggest that biotin supplementation, especially at high doses, should be avoided if not fully substantiated (as is the case in BD or HLCS deficiency, in BTBGD patients, and probably in SMVT-associated defects/deficiencies). Otherwise, e.g., in most dermatological disorders, carefulness is required, since much more information should be accumulated before reaching solid conclusions on the conditions under which biotin supplements are really needed, at what concentrations, and for what population groups they may be beneficial.

In summary, despite the abundant literature currently available, mechanisms of biotin homeostasis and their relation to pathogenesis and/or treatment of human disorders still deserve further investigation, at basic research and clinical level.

## Figures and Tables

**Table 1:** Biotinidase deficiency: recently reported cases.

Main Symptoms	Age at Diagnosis	Laboratory Methods Contributing to Diagnosis (Sample Analysis)	Dose of Biotin	Patient’s Response	Refs.
Neurological (optic neuropathy and myelopathy)	19-year-old	Biotinidase activity; biotinidase gene testing	20 mg/day	Significant improvement	[50]
Neurological (spinal cord demyelination, tetraparesis); dermatological (cutaneous manifestations)	2.5-year-old	Biotinidase activity; metabolite analysis	1 mg/kg/day	Marked improvement in neurological deficit Resolution of cutaneous manifestations	[51]
Neurological (motor regression with encephalopathy)	14-year-old	Biotinidase levels; biotinidase gene testing; metabolite analysis (lactate)	20 mg/day	Improvement	[52]
Neurological (anterograde manifestations, paraparesis; infrequent case of isolated bilaterally fornix infarction)	20-year-old	Biotinidase activity; biotinidase levels	High dose; patient paid no much attention to the treatment	No exact information; focus put on the patient’s unusual clinical presentation	[53]
Neurological	~5-month-old	Biotinidase activity; biotinidase gene testing; metabolite analysis	Initially: 7.5 mg/twice a day; subsequent slight changes in dose	Improvement of muscular hypotonia, prevention of epileptic seizures, satisfactory performance in deep tendon complexes; failure on poor feeding and intellectual disability	[54]
Neurological (longitudinally extensive transverse myelitis, optic neuritis, mimicking neuromyelitis optic spectrum disorder)	9-year-old	Biotinidase activity; biotinidase gene testing; metabolite analysis (lactate)	200 mg/day	Improvement	[55]
Dermatological (cutaneous lesions and sparseness of scalp hair); Neurological (hearing impairment)	4-year-old	Biotinidase levels; metabolite analysis (increased lactate)	20 mg/day	Dramatic improvement in cutaneous lesions/scalp hair; no effect on the auditory problems	[56]
Neurological; dermatological (quadriparesis with skin changes, non-compressive myelopathy)	6-year-old	Biotinidase activity; biotinidase gene testing; metabolite analysis (increased lactate and pyruvate)	Initially: 40 mg/twice a day After two weeks: 10 mg/twice a day	Full recovery	[57]
Neurological; respiratory distress	1-year-3-month-old	Biotinidase activity; biotinidase gene testing; metabolite analysis (lactate)	10 mg/day	Dramatic improvement	[58]
Neurological; dermatological	3-year-old	Biotinidase activity; biotinidase gene testing; metabolite analysis (increased lactate, 3-hydroxy-isovalerate)	40 mg/day	Improvement of neurological abnormalities; resolution of skin and scalp lesions	[59]
Neurological (leukoencephalopathy)	63-year-old	Biotinidase activity; metabolite analysis	High dose	Death (a few days after biotin treatment had started)	[60]
Neurological (progressive optic atrophy)	49-year-old	Biotinidase activity; biotinidase gene testing	10 mg/day	Improvement in peripheral vision; significant subjective improvement	[61]
Neurological (progressive optic atrophy, hearing difficulty)	13-year-old	Biotinidase levels; Biotinidase gene testing	40 mg/day	Initial symptoms remained stable; no new symptoms appeared	[62]
Neurological (paraparesis and bilateral vision loss)	13-year-old	Biotinidase activity; biotinidase gene testing	100 mg/day	Improvement	[63]
Neurological (spinal cord demyelination)	17-month-old	Biotinidase activity; biotinidase gene testing; metabolite analysis	20 mg/day	Striking improvement in many symptoms	[64]
Dermatological; neurological (acrodermatitis enteropathica-like skin eruption with neonatal seizures)	8-year-old	Biotinidase levels	10 mg/day	Full clearance of dermatological lesions	[65]
Neurological (lower limb spasticity, optic atrophy)	41-year-old	Biotinidase activity; biotinidase gene testing; metabolite analysis	High dose	Main symptoms remained stable with slight improvement of sensory deficits	[66]
Patient 1: Neurological (neuroimaging findings resembling neuromyelitis optica spectrum disorder)	26-month-old	Biotinidase enzyme activity; biotinidase gene testing; metabolite analysis	10 mg/three times a day	Normal neurodevelopmental profile	[67]
Patient 2: Respiratory (noisy breathing, recurrent choking episodes respiratory infections)	21-month-old	Biotinidase activity; biotinidase gene testing; metabolite analysis	10 mg/three times a day	No respiratory symptoms	[67]
Dermatological (hyperkeratosis, eczema, sparse yellowish white hair)	11-year-old	Biotinidase activity; biotinidase gene testing; metabolite analysis	20 mg/day	Dermatitis subsided, hair loss was resolved, and hair color was almost normal	[68]

**Table 2:** Biotin–thiamine-responsive basal ganglia disease: recent case reports.

Type of Symptoms	Age at Diagnosis	Laboratory Methods Contributing to Diagnosis (Sample Analysis)	Dose of Biotin	Dose of Thiamine	Patient’s Response	Refs.
Neurological (oculogyric crisis)	6-year-old	Gene testing	10 mg/kg/day	75 mg/kg/day	Significant improvement	[93]
Neurological (dystonia, tremor, slurred speech; the patient had accidentally ingested ~20 mL of ethyl alcohol)	2.5-year-old	Gene testing	50 mg/twice a day (8 mg/kg/day)	300 mg/three times a day (75 mg/kg/day)	Dramatic improvement	[94]
Neurological (visual blurriness, paresthesia, restless legs, memory difficulties)	16-year-old (first presentation); 17.5-year-old (diagnosis)	Gene testing	20 mg/day (~0.4 mg/kg/day)	600 mg/day (~12 mg/kg/day)	Many symptoms improved without therapy; upon therapy, most residual neurological symptoms were resolved	[95]
Neurological (irritability, opisthotonos, abnormal eye movements, axial hypotonia, mild gross motor delay, intermittent right esotropia)	2-month-old	Gene testing	10 mg/kg/day	20 mg/kg day	Improvement in development	[96]
Neurological (convulsions, confusion, bilateral pupillary light reflex delays, hypertonia of limbs, and brisk tendon reflexes of the limbs)	4-month-old first presentation); 9-month-old (diagnosis)	Gene testing	90 mg/day	400 mg/day.	Serious neurological dysfunction, despite initial apparent improvement; repeated pulmonary infections led to death from pneumonia (23-month-old)	[97]
Neurological (severe spasticity, increased muscle tone, hyperreflexia, dysarthria, dysphagia, ophthalmoplegia, truncal dystonia)	2-year-10-month-old	Gene testing	10 mg/kg/day	100 mg/twice a day)	Normal physical and neurological status with only mild reduced sensation on fingers	[98]
Neurological (sudden-onset feeding difficulty, impaired consciousness, encephalopathy)	6-year-old	Gene testing	10–15 mg/kg/day	10–20 mg/kg/day	Eye-tracking was regained, no recurrence of encephalopathy was observed, but other severe symptoms remained unresolved	[99]
Neurological (12-year history of hospitalizations for seizure disorders with recurrent encephalopathy episodes)	20-year-old	Gene testing	High-dose	High-dose	Death (one month after discharge, due to unknown causes)	[100]
Neurological (transient episodes of diplopia, multiple episodes of right focal seizures, abnormal posturing of the head and body, cognitive impairment, seizures, hypersomnolence, ataxia, generalized dystonia)	49-year-old	Gene testing	40 mg/day	125 mg/day	Significant improvement	[101]
Neurological (acute-onset encephalopathy); symptoms of diaphragmatic flutter (new-onset jerky abdominal movements)	9-year-old	No genetic test was performed to confirm clinical diagnosis	High dose	High dose	Diaphragmatic flutter was resolved	[102]
Patient 1: Neurological (excessive sleepiness, inability to walk, ptosis, irritability, and tonic posturing of the extremities)	2-year-old	Gene testing	High dose	High dose	MRI abnormalities resolved except for the central area of necrosis in the basal ganglia	[103]
Patient 2: Neurological (hypoactivity, drowsiness, seizures, unsteadiness, and frequent falls)	4-year-7-month-old	Gene testing	High dose	High dose	MRI abnormalities resolved except for the central area of necrosis in the basal ganglia	[103]
Patient 3: Neurological (unsteadiness and ptosis)	2-year-old	Gene testing	High dose	High dose	MRI abnormalities resolved except for the central area of necrosis in the basal ganglia	[103]
